# miR-421 and miR-30c Inhibit *SERPINE 1* Gene Expression in Human Endothelial Cells

**DOI:** 10.1371/journal.pone.0044532

**Published:** 2012-08-31

**Authors:** Alexandre Marchand, Carole Proust, Pierre-Emmanuel Morange, Anne-Marie Lompré, David-Alexandre Trégouët

**Affiliations:** 1 UMR_S 956, INSERM, Paris, France; 2 Faculté de Médecine, Université Pierre et Marie Curie, Paris, France; 3 ICAN Institute for Cardiometabolism and Nutrition, Paris, France; 4 UMR_S 937, INSERM, Paris, France; 5 UMR_S 1062, INSERM, Marseille, France; 6 Faculté de Médecine, Université de la Méditerranée, Marseille, France; Florida State University, United States of America

## Abstract

In this work, we assessed whether *SERPINE1* expression could be under the influence of microRNAs (miRNAs) predicted to bind the *SERPINE1* 3′UTR region. We specifically focused on the 3′UTR region harboring a common polymorphism, rs1050955, that have been found associated to *SERPINE1* monocyte expression, and investigated whether the presence of different alleles at rs1050955 could modify the miRNAs binding efficiency and affect PAI-1 protein levels. We demonstrated that, in human umbilical vein endothelial cells, both miR-421 and miR-30c directly interacted with PAI-1 mRNA to inhibit the expression of the associated protein. However, these inhibitory mechanisms were independent on the allele present at the rs1050955 locus. We further showed that miR-421 levels correlated with PAI-1 activity in the plasma sample of 40 patients with venous thrombosis. Our results strongly suggest that the regulation of PAI-1 molecule could be under the influence of several miRNAs whose measurement in the plasma of patients could be envisaged as a biomarker for inflammatory and thrombotic disorders.

## Introduction

Plasminogen Activator Inhibitor-1 (PAI-1) is a key target in the etiologic and mechanistic study of metabolic syndrome and diseases arising from it. PAI-1 is the primary inhibitor of both tissue and urokinase type plasminogen activators and, as such, the main inhibitor of the fibrinolytic system.

Several groups, including ours [1], [2], [3], have hypothesized that by preventing blood clot lysis, PAI-1 participates in the development of athero and venous thrombotic disorders, a hypothesis that has been supported by several large epidemiological studies (reviewed in [4]). As recently reviewed in Iwaki et al [5], PAI-1 is also involved in the physiopathology of non-thrombotic disorders making the disentangling of its underlying regulatory mechanisms a matter of great interest.

PAI-1 plasma levels are known to be influenced by both environmental and genetic factors, the latter hypothesized to explain about 30% of their variability [6]. However, the exact molecular mechanisms underlying these genetic determinants are poorly understood. Up to very recently, only the *SERPINE1* gene that codes for the PAI-1 protein was identified to harbor single nucleotide polymorphisms (SNPs) influencing PAI-1 levels, some of these SNPs being besides variants associated with clinical outcome [7], [8]. A recent Genome-Wide Association Study (GWAS) conducted in 1,093 individuals from four distinct ethnic groups failed to identify novel robust loci associated with PAI-1 variability [9]. This strongly suggests that a substantial part of the genetic variability remains to be characterized. By interrogating the public GHS_express database [10] reporting the results of a genome wide search for SNPs influencing monocyte gene expressions in a sample of 1,467 healthy individuals, we observed that the rs1050955 mapping the *SERPINE1* 3′UTR region explained ∼4% (p<10^−12^) of the *SERPINE1* monocyte expression, the rare rs1050955-A allele being associated with decreased *SERPINE1* expression.

MicroRNAs (miRNAs) are small 18–24 bp endogenous non coding RNAs that inhibit mRNA translation either via mRNA degradation or via repression of mRNA translation [11]. It has been shown that nucleotides 2 to 8 of the miRNA, called “seed sequence” are essential and sufficient to promote translation inhibition of a target mRNA although a higher sequence complementarity is necessary to induce mRNA clivage [12], [13]. Recently *SERPINE1* expression was shown to be regulated by miR-30c and miR-301a in pulmonary endothelial cells [14]. According to bioinformatics tools such as Targetscan [15] and microSniper [16], the rs1050955 polymorphism is predicted to map in the close vicinity of target binding sites for several miRNAs including hsa-miR-300, hsa-miR-381, hsa-miR-548l, hsa-let7f-1*, has-let7-a* and hsa-miR-421. The recent observations that miRNA fixation could be modified by only one SNP in its binding sequence and could subsequently modify protein expression [17], [18] has led us to hypothesize that *SERPINE1* rs1050955 could exert a functional effect on the associated expression through a modulation of miRNA's binding efficiency. The first objective of our study was to determine whether *SERPINE1* expression could be under the influence of some of these miRNAs predicted to bind near the polymorphism. Second, as experimental works have demonstrated that the presence of a polymorphism could disrupt or create a miRNA target binding site and influence the expression of the targeted mRNA [17], [19], [20], we investigated whether the rs1050955 could modify miRNAs binding efficiency and affect SERPINE1 protein levels.

## Results

**Figure 1:**
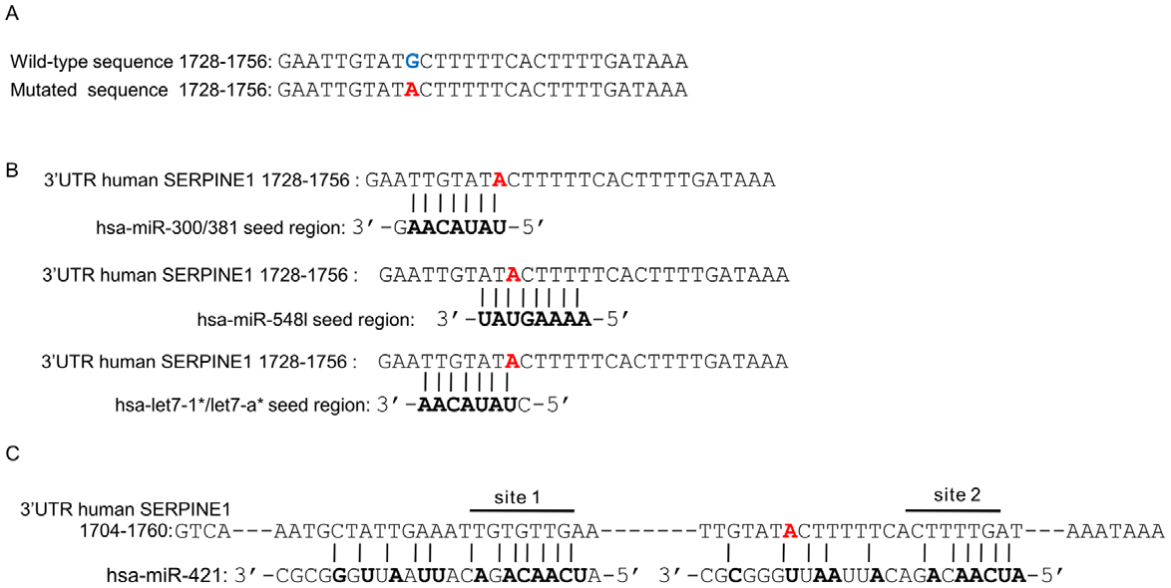
miRNA binding sequences in the *SERPINE1* 3′UTR region. The 3′UTR human *SERPINE1* sequence is 1841 bp long. The rs1050955-A muted allele is shown in red bold. **A.** *SERPINE1* DNA sequence showing the rs1050955 G/A polymorphism in position 1737. **B.** Representation of various miRNA seed region and their complementary sequence in *SERPINE1* 3′UTR 1728–1756 region. **C.** Alignment of miR-421 onto *SERPINE1* 3′UTR 1704–1760 region which includes two predicted binding sites, site 1 and site 2, complementary to the miR-421 seed sequence.

According to bioinformatics predictions [15], [16], compared to the wild rs1050955-G allele, the rs1050955-A allele allows a better match with the seed sequence of various miRNAs (hsa-miR-300, hsa-miR-381, hsa-miR-548l, hsa-let7f-1*, has-let7-a*). Moreover in humans, hsa-miR-421 was predicted to bind close to the polymorphism sequence (this binding sequence was referred to as site 1 in this work) although this site was not conserved among species. Interestingly a second binding site for hsa-miR-421 according to sequence similarity (called site 2) was found 23 bp further. The polymorphism rs1050955 allows a better base pairing for hsa-miR-421 on this site 2 although not on its seed sequence (**Figure 1**).

These six miRNAs were tested for co-expression with PAI-1 in a variety of cell lines in which the later is known to be expressed, including human acute monocytic leukemia cells (THP1), human mammary epithelial cells (HMEC), human aortic endothelial cells (HAEC) and human umbilical vein endothelial cells (HUVEC). While hsa-miR-548l, hsa-miR-300, has-miR-381, has-let7f-1* and hsa-let7a* miRNAs were not or very poorly expressed in any of the aforementioned cell lines, both PAI-1 (see **Table 1**) and miR-421 (see **Table 2**) were homogeneously found expressed in these cell lines with the highest levels observed in HUVEC. As a consequence, we pursued our investigations on the influence of rs1050955 on miR-421's binding to *SERPINE1* 3′UTR region and on *SERPINE1* expression in HUVEC.

**Table 1:** Detection of PAI-1 mRNA by qRT-PCR in various cell lines.

	Ct values(1)		Expression(2)
Cell lines	PAI-1	RPL32	
HUVEC	17.87	21.11	1.00
HMEC	19.09	20.41	0.26
HAEC	16.56	20.58	1.72
THP1	26.17	19.63	0.0011

(1) Cycle Threshold (Ct) values.

(2) Relative transcript expression levels were calculated by use of the the 2^−ΔΔCT^ method using HUVEC as reference.

**Table 2:** Detection of miRNAs of interest by qRT-PCR in various cell lines.

	Ct values(1)									Expression(2)	
Cell lines	miR-300	miR-381	miR-548l	let-7a*	let-7f-1*	U6	miR-421	miR-30c	U6	miR-421	miR-30c
HUVEC	34.74	32.17	36.57	32.47	32.77	30.86	26.81	22.79	30.86	1.00	1.00
HMEC	35.40	30.95	35.33	31.36	31.60	30.92	28.05	22.69	30.92	0.44	1.12
HAEC	38.55	35.80	37.51	31.47	31.47	30.12	28.79	23.93	30.12	0.15	0.27
THP1	34.62	34.87	36.18	33.94	32.77	31.22	28.03	24.65	31.22	0.55	0.35

(1) MiRNAs with Cycle Threshold (Ct) values over 31 were considered not sufficiently expressed for further investigations.

(2) Relative transcript expression levels were calculated by use of the the 2^−ΔΔCT^ method using HUVEC as reference.

**Figure 2:**
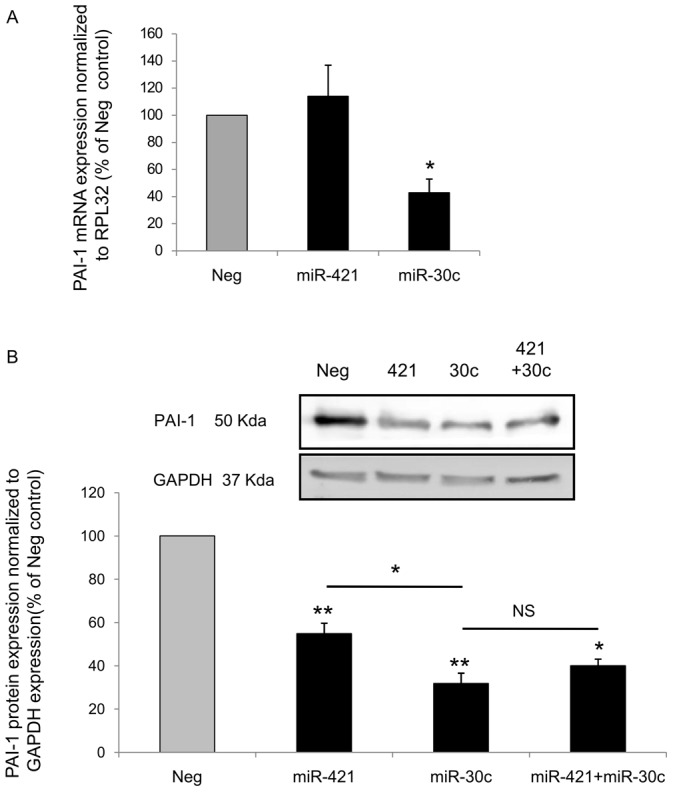
MiR-421 and miR-30c inhibit *SERPINE1* expression in HUVEC. **A.** Quantification by qRT-PCR of PAI-1 mRNA level after over-expression of either Pre-miR-421, Pre-miR-30c or a Pre-miR Negative control (Pre-Neg) in HUVEC cells. RPL32 mRNA level was used for normalization and data shown were expressed as percentage compared to Negative control (*, p<0.05 n = 4). **B.** Western-Blot and quantification of PAI-1 and GAPDH protein level after over-expression of either Pre-miR-421, Pre-miR-30c or both compared to Pre- Neg transfected cells. Data shown were normalized to GAPDH protein level and expressed as percentage compared to Negative control (n = 5 for miR-421 and miR-30c, n = 3 for miR-421+30c; *, p<0.05; **, p<0.01).

At a first step, we over-expressed miR-421 by transfection of 3 nM of dedicated miRNA precursor (Pre-miR-421) and checked whether this was accompanied by a decrease in PAI-1 mRNA level or of PAI-1 protein translation. MiR-421 overexpression was not followed by any modulation of PAI-1 mRNA level compared to a negative control (Pre-Neg) (**Figure 2A**). Conversely, the over-expression of miR-30c previously shown to inhibit PAI-1 mRNA level in pulmonary endothelial cells [14] was associated with a 43% decrease in PAI-1 mRNA level (**Figure 2A**). However, as shown in **Figure 2B**, both over-expression of miR-421 and miR-30c were associated with a significant decrease in PAI-1 protein level, 59% and 32%, respectively. Note that simultaneously co-expressing miR-421 and miR-30c did not reveal any additive effect on PAI-1 inhibition (**Figure 2B**).

**Figure 3:**
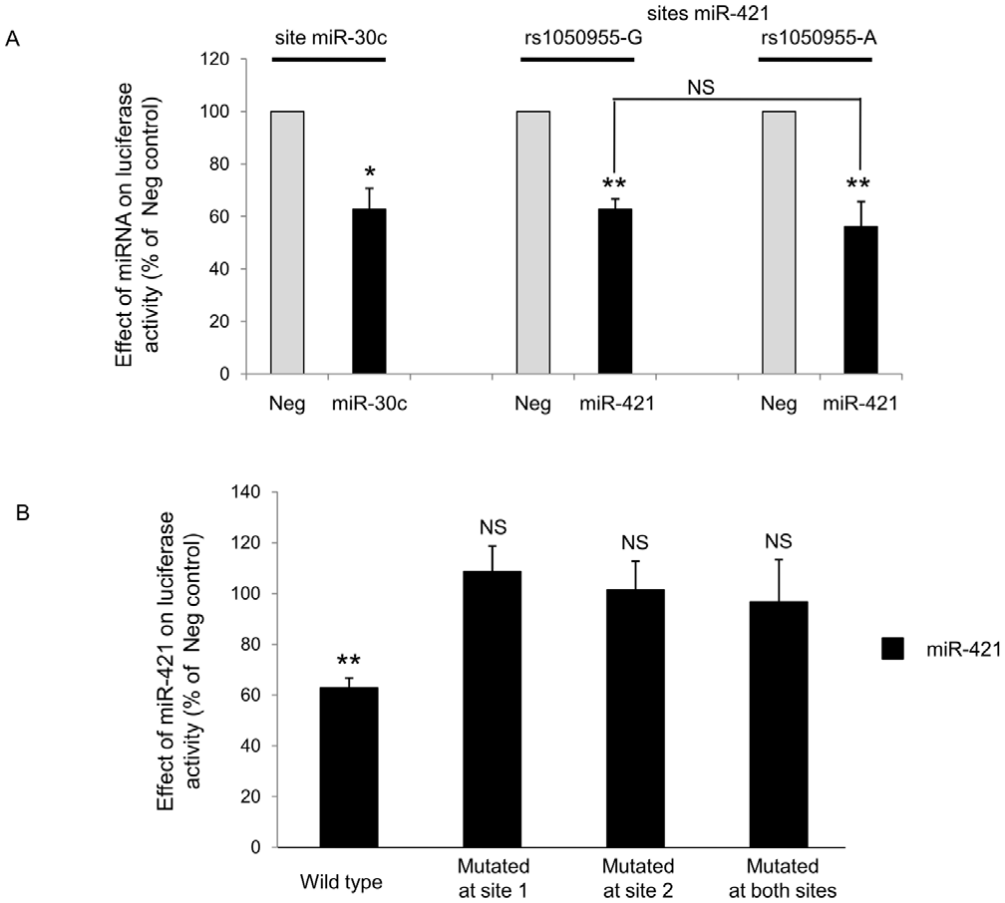
Influence on luciferase activity of miR-421 and miR-30c binding to the 3′UTR *SERPINE1* 1704–1760 region. **A.** psicheck2 vector containing 3′UTR *SERPINE1* sequence surrounding miR-30c predicted binding site or miR-421 predicted binding sites according to the allele present at rs1050955 fused to renilla luciferase were co-transfected with Pre-Neg, Pre-miR-30c or Pre-miR-421. Graph shows renilla luciferase activity normalized to firefly luciferase activity and expressed as percentage of Pre-Neg transfected cells (n = 4 for miR-30c and n = 5 for miR-421; *, p<0.05; **, p<0.01)). **B.** Various 3′UTR *SERPINE1* sequence containing both miR-421 site 1 and site 2 predicted binding sites or mutation of each seed sequence binding sites were fused to renilla luciferase. Plasmids were transfected with Pre-Neg or Pre-miR-421. Graphs show renilla luciferase activity normalized to firefly luciferase activity and expressed as percentage of Pre-Neg transfected cells (n = 5 except for site1+2 1 mut 2 mut construct, n = 4; *, p<0.05; **, p<0.01).

The second stage consisted in checking whether the observed miR-421 inhibitory effect on PAI-1 protein was due to a direct interaction between the miRNA and the PAI-1 mRNA sequence. Sequences of the 3′UTR of the PAI-1 mRNA surrounding the two close miR-421 potential binding sites (site 1 and site 2 described in **Figure 1**) containing the wild-type (G) or the mutated (A) allele at rs1050955 were subcloned in a luciferase reporter vector, as well as constructions with mutated seed sequence of either site 1 or site 2 miR-421 binding sites. MiR-30c binding sequence was used as a positive control. Over-expression of miR-421 and miR-30c lead to a decrease in luciferase activity of 40% in both cases (**Figure 3A**), indicating that both miRNAs can directly bind to PAI-1 3′UTR mRNA. The presence of the rs1050955-A allele was not significantly associated with a higher luciferase activity decrease compared to the rs1050955-G allele (**Figure 3A**). Interestingly, the introduction of mutations in the sequence complementary to miR-421 seed sequence from site 1 or site 2 were sufficient to abolish the miR-421 sensitive decrease of luciferase expression, indicating that the two close binding sites are necessary to obtain an inhibitory effect by miR-421 (**Figure 3B**).

**Figure 4:**
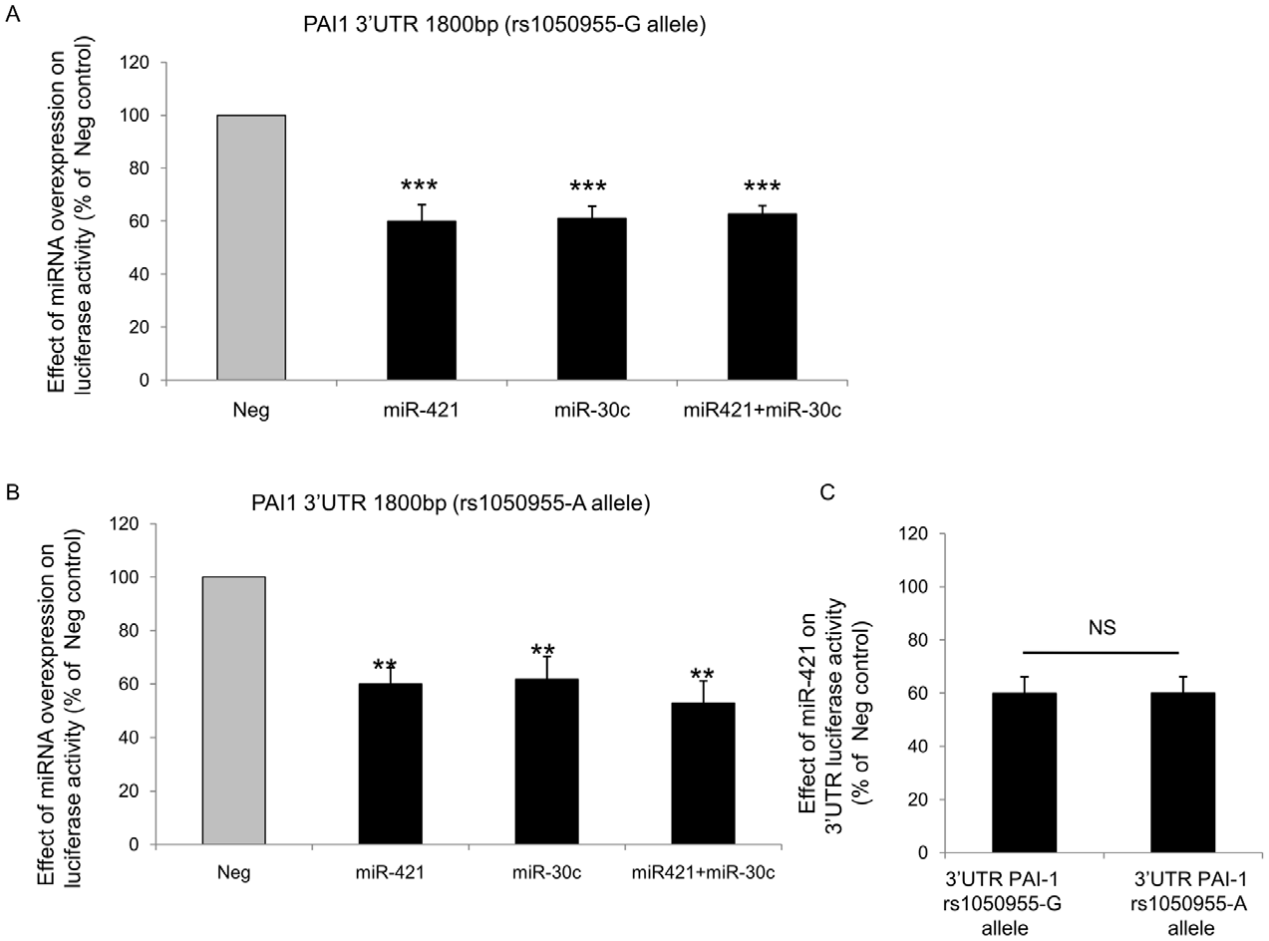
Influence of miR-421 and miR-30c binding to total 3′UTR *SERPINE1* on luciferase activity. **A.** Psicheck2 vector containing total 3′UTR *SERPINE1* sequence fused to renilla luciferase was co-transfected with Pre-Neg, Pre-miR-30c, Pre-miR-421 or both Pre-miR-30c and Pre-miR-421. Graph shows renilla luciferase activity normalized to firefly luciferase activity and expressed as percentage of Pre-Neg transfected cells (n = 6, p<0.005,***). **B.** Psicheck2 vector containing total 3′UTR *SERPINE1* sequence with the mutated rs1050955-A allele fused to renilla luciferase was co-transfected with Pre-Neg, Pre-miR-421, Pre-miR-30c or both. Graph shows renilla luciferase activity normalized to firefly luciferase activity and expressed as percentage of Pre-Neg transfected cells (n = 5 ** p<0.01). **C.** Comparison of miR-421 inhibitory effect on luciferase activity between *SERPINE1* 3′UTR wild-type or mutated at rs1050955 (n = 4).

*SERPINE1* 3′UTR is 1841 bp long and miR-30c and miR-421 binding sites are separated by more than 1000 bp (662–668/1722–1729 and 1746–1752 for sequence complementary to seed sequence of miR-30c or miR-421 respectively). As both miR-421 and miR-30c were expressed in HUVEC, we cloned the entire PAI-1 3′UTR in the luciferase expression vector and over-expressed these two miRNAs. A homogeneous decrease in luciferase expression was observed when miR-30c and miR-421 were over-expressed (63% and 60%, respectively). The joint co-expression of both miRNAs led to a similar (63%) decrease (**Figure 4A**). The presence of the rs1050955-A allele SNP in the molecular construct did not show specific effect as it was associated with a 53% decrease of luciferase expression (**Figure 4B–C**).

**Figure 5:**
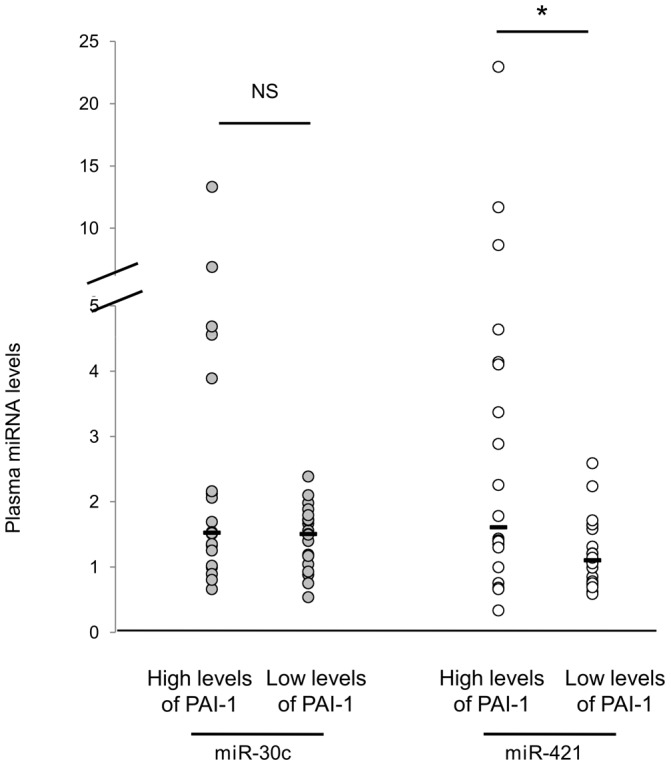
Plasma levels of miR-421 and miR-30c in plasma samples of venous thrombosis patients. MiR-421 and miR-30c were detected by qRT-PCR in plasma samples from two groups of 20 patients either with low (1.6+/−1 ui/ml) or high (40.5+/−13 ui/ml) PAI-1 plasma levels. 40 fmol of synthetic cel-miR (39/54/238) were used for normalization. Median values are shown in black line (*: p<0.05).

Finally, we measured both miR-421 and miR-30c levels in the plasma of 40 venous thrombosis patients including 20 patients with high (mean 40.50±13.05 UI/ml) and 20 patients with extremely low (mean 1.63±0.99 UI/ml) PAI-1 plasma levels. Levels of miR-421 and of miR-30c were strongly correlated, the corresponding Spearman correlation coefficient being 0.51 and 0.66 in the low and high PAI-1 groups, respectively. However, while no difference in miR-30c levels was observed between the “low” and “high” groups (1.46 vs 2.71, p = 0.327), miR-421 was significantly (p = 0.024) decreased in patients with low PAI-1 compared to patients with high PAI-1 levels (1.22 vs 3.81) (**Figure 5**). It is noteworthy that the miR-30c and miR-421 plasma variability tended to be higher in patients with high PAI-1 levels than in patients with extremely low levels of PAI-1.

## Discussion

By interrogating a public database reporting the results of a genome-wide survey for SNPs influencing monocyte gene expressions [10], we observed that the *SERPINE1* rs1050955 explained about 4% of the variability of its associated gene. This G/A SNP is located in the 3′UTR region of the *SERPINE1* gene encoding for the PAI-1 molecule, and is predicted to reside at target binding sites for several miRNAs. This lead us to hypothesize that *SERPINE1* gene expression could be influenced by miRNA-sensitive mechanisms dependent on the allele present at the rs1050955 locus.

Among the miRNAs predicted to bind the *SERPINE1* region where rs1050955 lies, we demonstrated that only the miR-421 was jointly expressed with *SERPINE1* in a variety of cell types. MiR-421 was further shown to directly interact with PAI-1 mRNA to inhibit the expression of the associated protein in HUVEC. We demonstrated that mir-421 binds to two sites and that its inhibitory effect is abolished as soon as the seed sequence of at least one of these sites is mutated. This could suggest the existence of cooperative effect or of a required secondary structure that are affected when seed sequences are mutated. However, our work did not provide support to our starting hypothesis about the role of the rs1050955 on *SERPINE1* expression as we did not observe any evidence for a modulation of miR-421's binding efficiency according to the allele present at the rs1050955. Possible explanations for the discrepancy between our experimental results and the reported association in the GHS database include – the existence of another yet unidentified miRNA whose binding efficiency depends on this polymorphism, – rs1050955 exercising its effect on the local secondary structure and structural accessibility of the mRNA [21], on the mRNA half-life as it has been shown when mutations occur in or near AU-rich elements in the 3′UTR [22], or on the binding of transacting factors could be affected [22]. As it can not be ruled out that the reported association was a statistical artefact, further experiments are needed to investigate these hypotheses.

Nevertheless, we further observed an association of plasma levels of miR-421 with those of PAI-1 in a sample of 40 patients with venous thrombosis. Indeed, miR-421 plasma levels were found increased in patients with very high levels of PAI-1 compared to patients with extremely low PAI-1 levels. This positive correlation between PAI-1 and miR-421 plasma levels could be considered as intriguing compared to the negative correlation we observed on the corresponding expressions in HUVECs. However it should be noted that many cell types could be responsible for PAI-1 (e.g. endothelial cells, hepatocytes, monocytes and platelet) and miR-421 plasma expression. High miRNA plasma levels are not always a sign of cellular damage. There is emerging evidence suggesting that some miRNAs could be actively secreted or on the contrary selectively retained in the cells. Moreover they could represent a new mechanism for intercellular communication and exert their action in cell types or tissues different from their site of synthesis [23]. High miR-421 plasma levels could thus be an adaptative response to high PAI-1 levels or a sign of an inflammatory response.

Conversely, it could also be hypothesized that plasma circulating levels of miR-421 could reflect free miR-421 that had not bound to PAI-1 mRNA. They should then be decreased in subjects whose PAI-1 levels have been lowered by an efficient miRNA-binding mechanism. Distinguishing between these hypotheses deserves further experimental investigations. Nevertheless, our work strongly suggests that miR-421 participates to the regulatory control of *SERPINE1* expression.

MiR-421 binding sites on PAI-1 3′UTR are poorly conserved among species but we showed that this miRNA has indeed a true inhibitory effect on PAI-1 in humans. This underlines the risk of missing true miRNA-sensitive regulatory mechanisms if one focus only on miRNAs that are conserved across all species. Little is known about miR-421 and its pathophysiological role in human diseases. It has recently been described as oncogenic miRNA overexpressed in pancreatic- [24], gastric- [25] or hepato-carcinoma [26] cancers. In pulmonary artery smooth muscle cells, miR-421 was found inducible by transforming growth factor beta (TGFb) and bone morphogenetic protein 4 (BMP4) via a conserved Smad binding element (SBE) – like sequence [27]. Because such SBE sequence is also present in the *SERPINE1* promoter region [28] and because TGFb is a major regulator of PAI-1 [29], our results would suggest that the TGFb signaling pathway allows a fine tuning of *SERPINE1* expression by controlling its up- and down- regulation.

Our work also extends recent results obtained in pulmonary endothelial cells [14] and adipose tissue [30] as we demonstrated that miR-30c also participate to PAI-1 expression in HUVECs. Consistent with aforementioned results, we indeed showed that miR-30c inhibits both PAI-1 mRNA and protein levels. The mRNA inhibition was not observed with miR-421 and this could explain why the inhibitory effect on PAI-1 protein was stronger with miR-30c than with miR-421. MiR-421 and miR-30c overexpression did not show any additive effect on PAI-1 protein expression. This could be explained by the degradation of *SERPINE1* mRNA by miR-30c before miR-421 could exert its inhibitory effect.

Conversely, unlike miR-421, plasma levels of miR-30c were not associated with PAI-1 in our sample of venous thrombosis patients despite a trend for such association. Several limitations must be acknowledged. First, our starting hypothesis was derived from results conducted on circulating monocytes [10]. Here we focused on endothelial cells because these cells play a key role in the pathophysiological mechanisms related to PAI-1 such as inflammation, obesity and thrombosis [31], [32]. Second, we have used patients with venous thrombosis to investigate the association of miR-421 and miR30-c with PAI-1 plasma levels. Obtained results may not be generalized to other disease patients. This may explain why we did not observe an association of miR-30c with PAI-1 as initially reported in sickle anemia diseased patients [14]. The low sample size of our study could also have limited the power for detecting a differential miR30-c expression.

In conclusion, we demonstrated for the first time that miR-421 participates to the regulation of PAI-1 molecule. Our work also showed that miR-421 can be efficiently measured in plasma fluid leading promising perspectives for assessing the role of miR-421 as a biomarker for inflammatory and thrombotic disorders.

## Materials and Methods

### Ethics Statement

Informed written consent from the participating individuals was obtained in accordance with the Declaration of Helsinki and ethics approval were obtained from the “Departement santé de la direction générale de la recherche et de l'innovation du ministère” (Projects DC: 2008–880 & 09.576).

### Bioinformatic analysis

MiRNAs predicted to bind to *SERPINE1* 3′UTR in particular in the region surroundind the rs1050955 polymorphism were predicted using microSNiPer [16], Targetscan release 6.1 [15] and miRANDA algorithms [33]. Sequence complementary to miR-421 seed sequence were also directly searched along serpine1 3′UTR.

### Cell culture

Human umbilical vein endothelial cells (HUVEC) were isolated as described by *Jaffe et al* [34]. and used at passage 2–5. HUVECs were cultured in MCDB 131 Medium (Gibco, Invitrogen, Cergy-Pontoise, France) supplemented with 10% heat-inactivated fetal calf serum (Biowest, Nuaillé, France). Cell cultures were maintained in a humidified 95% air–5% CO2 incubator at 37°C. Other cell lines used in the study to detect PAI-1 and miR-421 expression included human mammary epithelial cells (HMEC), human aortic endothelial cells (HAEC), human acute monocytic leukemia cells (THP1), and fresh monocytes preparation.

### MiRNA overexpression

On the day of transfection, HUVEC cells at 80% confluence in 6-well plates were washed twice in PBS and antibiotic-free medium was added. Then cells were transfected either with 3 nM Pre-miR™ miRNA Precursor for miR-421 (Pre-421) or Pre-30c or Negative control (Pre-Neg) (Life Technologies, Villebon sur Yvette, France) with Lipofectamine transfection reagent (Life Technologies) according to the manufacturer's recommandations: 300 µl of Opti-MEM+Pre-miR+3 µl Lipofectamine were added to the cells. 24 hours later cells were washed twice and then cultured for 2 days in 10% serum MCDB 131 Medium before extraction of RNA and proteins.

### RNA isolation and quality measurement

Total RNA including miRNAs from cultured HUVEC were isolated with the *mir*Vana miRNA isolation kit (Life technologies) following the manufacturer's instructions. RNA concentrations were determined with a Nanodrop ND-1000 spectrophotometer (Thermo Fisher Scientific, Courtaboeuf, France). RNA quality was determined using the RNA nano LabChip ^®^ and a 2100 Bioanalyzer (Agilent Technologies, Massy, France). Only samples with an RNA integrity number higher than 8 (as determined by the Bioanalyzer) were accepted for the following studies.

### RT-PCR

Total RNA Reverse transcriptase-PCR analysis was performed as described elsewhere [35], using the Absolute QPCR SYBR green mix (ABgene, Courtaboeuf, France) on an MX3005P QPCR system (Stratagene, Agilent Technologies, Massy, France). Primer pairs used in this study (see **Table 3**) were checked by using a standard curve in order to ensure that the reaction efficiency was between 90% and 110%. Melting curves were analysed to check the specificity of each RT-PCR amplification. Transcript levels were normalized to the RPL32 mRNA content, and the relative transcript level between two samples was calculated by using the 2^−ΔΔCT^ method.

**Table 3:** Oligonucleotides used in this study.

PAI-1 mRNA quantification	Human-PAI-1-f:	CCGGAACAGCCTGAAGAAGTG
	Human-PAI-1-rev:	GTGTTTCAGCAGGTGGCGC
	Human-RPL32-f:	CCCAAGATCGTCAAAAAGA
	Human-RPL32-rev:	TCAATGCCTCTGGGTTT
Luciferase 3′UTR assays	XhoI- 3′UTR serpine1 miR-421sites1+2-f:	tcgagGGTGTCAAATGCTATTGAAATTGTGTTGAATTGTATGCTTTTTCACTTTT*G*ATAAATAAAgc
	NotI-3′UTR serpine1 miR-421 sites1+2-rev:	ggccgcTTTATTTATCAAAAGTGAAAAAGCATACAATTCAACACAATTTCAATAGCATTTGACACCc
	XhoI- 3′UTR serpine1MUTSNP -miR-421-f:	tcgagGGTGTCAAATGCTATTGAAATTGTGTTGAATTGTATACTTTTTCACTTTTGATAAATAAAgc
	NotI-3′UTR serpine1MUTSNP -miR-421-rev:	ggccgcTTTATTTATCAAAAGTGAAAAAGTATACAATTCAACACAATTTCAATAGCATTTGACACCc
	XhoI- 3′UTR serpine1 miR-30c-f:	tcgagATTTTGGAGTGTAGGTGACTTGTTTACTCATTGAAGCAGATTTCTGCgc
	NotI-3′UTR serpine1 miR-30c-rev:	ggccgcGCAGAAATCTGCTTCAATGAGTAAACAAGTCACCTACACTCCAAAATc
	XhoI- 3′UTR serpine1 miR-421 site 1 mut-f:	tcgagGGTGTCAAATGCTATTGAAATTGGTGGTCATTGTATGCTTTTTCACTTTT*G*ATAAATAAAgc
	NotI-3′UTR serpine1 miR-421site 1 mut-rev:	ggccgcTTTATTTATCAAAAGTGAAAAAGCATACAATGACCACCAATTTCAATAGCATTTGACACCc
	XhoI- 3′UTR serpine1 miR-421 site 2 mut-f:	tcgagGGTGTCAAATGCTATTGAAATTGTGTTGAATTGTATGCTTTTTCAGGTGGTCGAAATAAAgc
	NotI-3′UTR serpine1 miR-421 site 2 mut-rev:	ggccgcTTTATTTCGACCACCGTGAAAAAGCATACAATTCAACACAATTTCAATAGCATTTGACACCc
	XhoI- 3′UTR serpine1 miR-421 site 1mut+site 2 mut-f:	tcgagGGTGTCAAATGCTATTGAAATTGGTGGTCATTGTATGCTTTTTCAGGTGGTCGAAATAAAgc
	NotI-3′UTR serpine1 miR-421 site1 mut+site 2 mut-rev:	ggccgcTTTATTTCGACCACCGTGAAAAAGCATACAATGACCACCAATTTCAATAGCATTTGACACCc
Amplification of total *SERPINE1* 3′UTR	XhoI- 3′UTR serpine1 1800bp-f:	CGCCctcgagTGGGACAAAACTGGAGATGC
	NotI-3′UTR serpine1 1800bp-rev:	TTTTgcggccgcGACTGTCCTGACATATTCTTCGT
Introduction of rs1050955 A variant	3′UTR 1800bp +SNP-f:	TGTGTTGAATTGTATACTTTTTCACTTTTG
	3′UTR 1800bp+SNP-rev:	CAAAAGTGAAAAAGTATACAATTCAACACA

miRNA specific RT-PCR were performed using specific Taqman miRNA assays (Life technologies) and Taqman Universal PCR Master Mix, no AmpErase UNG according to the manufacturer's instructions and normalized to small RNA RNU6B (U6) probe for human samples. The relative transcript level between two samples was calculated by using the 2^−ΔΔCT^ method.

### Protein preparation and western blot

Protein extracts from HUVEC cells were prepared using Promokine Mammalian Whole Cell Extraction kit (PromoCell GMBH, Heidelberg, Germany). The total protein concentration was determined with the Bio-Rad assay (Bio-Rad, Marne-la-coquette, France). Equal amounts (50 µg) of total protein were separated by 10% SDS-PAGE followed by immunoblotting with a Trans-Blot Semi-Dry (Bio-Rad), following the manufacturer's protocol. The following antibodies were used: rabbit polyclonal antibody to GAPDH (ab9485, 1/2500) and mouse anti-human PAI-1 (ab82218, 1/1000) from Abcam (Paris, France). Western blots were revealed with the ECLplus reagent (GE Healthcare, Diegem, Belgique) and scanned with an Ettan-DIGE Imager (GE Healthcare). Densitometric analysis was performed with NIH Image/ImageJ, and the expression level of PAI-1 was normalized to GAPDH.

### Luciferase reporter constructs

PsiCHECK-2 vector (Promega, Charbonnieres, France) containing both firefly and renilla luciferase genes was used to introduce 3′UTR sequences immediately downstream the stop codon of the Renilla luciferase gene. Oligonucleotides corresponding to the serpine1 3′UTR surrounding the predicted miR-421 binding sites with or without mutation and miR-30c binding site were inserted into psiCHECK-2 using XhoI and NotI restriction enzyme (see **Table 3**).

Oligonucleotides forward and reverse were resuspended at 10 µM in 150 mM NaCl. After 5 minutes at 95°C, and 30 minutes at room temperature, annealed sequences (1 µl) were directly ligated into XhoI-NotI-digested plasmid (50 ng) using NEB Quick ligation kit (New England Biolabs, Evry, France) according to the manufacturer's instructions. Total 3′UTR (1800 bp) of PAI-1 was amplified from genomic DNA from HUVEC by PCR (35 cycles: 95°C 1 min-52°C 1 min-72°C 2 min), purified using Qiaquick Gel Extraction kit (Qiagen) and digested by XhoI and NotI before ligation into XhoI-NotI digested psiCHECK-2 vector. The G&
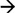
A SNP was also introduced in the 1800 bp 3′UTR fragment by two steps of PCR (see **Table 3** for oligonucleotides sequences). Finally 2 µl ligation were transfected into 50 µl DH5a cells (Life technologies). Plasmids were prepared from colonies grown on LB plates supplemented with Ampicilline and sequenced before further experiments.

### miRNA target validation by luciferase assay

MiRNA target reporter assays were performed in triplicate in 24-well plates. HUVEC at 80% confluence were washed twice with PBS+Ca+Mg (Life technologies) and 500 µl antibiotics-free medium was added per well. 200 ng of each target construct and 3 nM Pre-miR or Pre-Neg were mixed with 2 µl Lipofectamine in 100 µl Opti-MEM according to the manufacturer's protocol. After 24 hours, cells were washed twice in PBS+Ca+Mg and fresh medium was added. After 48h of incubation, Firefly and Renilla luciferase activities were sequentially measured using the Dual-Glo Luciferase Assay system (Promega). 100 µl of Dual-Glo luciferase reagent were added on each well and the plate was shaked for 30 minutes at 600 rpm to allow the lysis of the cells. 75 µl of cell lysate were transferred into a white 96-well Fluoronunc plate (VWR, Fontenay-sous-bois, France) to avoid side effects and luciferase activity was detected using Winglow PC Software and LB 96 microplate Luminometer (EG &G Berthold, Thoiry, France). Then 75 µl of Dual Stop and Glo reagent were added. After 10 minutes agitation at 600 rpm, renilla luciferase activity was detected. Renilla luciferase signals were normalized with Firefly luciferase signals for each well.

### Detection of PAI-1 and miRNA from human plasma samples

Plasma samples from patients with high or low PAI-1 expression were obtained from consecutive patients who came in December 2011 for thrombophilia screening at the Centre d'Exploration des Pathologies Hémorragiques et Thrombotiques (CEHT) of la Timone Hospital (Marseille, France). All patients had a personal history of venous thrombosis. Venous blood samples were collected in the morning after subjects had fasted for 12 hours. The blood was then centrifuged for 30 minutes at 2,000 g and 4°C, and the resulting plasma was collected and stored in aliquots at −70°C until assayed. PAI-1 activity levels were measured using the TriniLIZE PAI-1 activity kit from Trinity Biotech (Bray, Ireland). One unit of PAI-1 activity is defined as the amount of PAI-1 that inhibits one international unit of human single chain tPA as calibrated against the international standard for tPA. Inter-assay variation coefficients of pooled from 30 healthy volunteers were 10%.

Out of the 80 patients who came during the month of December 2011, we selected those with PAI-1 values in the 1^st^ and in the 4^th^ quartile of the distribution (ie 20 patients with low and 20 patients with high PAI-1 levels).

Extraction of miRNA from plasma was performed as previously described [36]. Briefly 300 µl of plasma were mixed with 5 volumes Qiazol (Qiagen). 40 fmol of Spike In control synthetic miRNA cel-miR-39, cel-miR-54, cel-miR-238 (synthesized by Eurogentec) were added and miRNeasy mini kit (Qiagen) was used to purify miRNA, which were eluted in 30 µl H2O. 5 µl were used for each RT reaction with specific Taqman miRNA primers (Life Technologies). Normalization was performed using Spike In cel-miRs Ct values as internal controls as previously described [37].

### Statistical Analysis

Data were expressed as means ± SEM. Two-groups comparison was performed using the non parametric Mann-Whitney test. A p-value threshold of 0.05 was used to declare statistical significance
